# First evidence of underwater vocalisations in hunting penguins

**DOI:** 10.7717/peerj.8240

**Published:** 2019-12-18

**Authors:** Andréa Thiebault, Isabelle Charrier, Thierry Aubin, David B. Green, Pierre A. Pistorius

**Affiliations:** 1 DST/NRF Centre of Excellence at the Percy FitzPatrick Institute of African Ornithology, Institute for Coastal and Marine Research, Department of Zoology, Nelson Mandela University, Port Elizabeth, South Africa; 2 CNRS UMR 9197, Institut des Neurosciences Paris-Saclay, Université Paris Sud, Orsay, France

**Keywords:** Bioacoustics, Biologging, Foraging, Feeding, Seabirds, Spheniscidae, Penguin, Underwater vocalisation, Marine predators

## Abstract

Seabirds are highly vocal on land where acoustic communication plays a crucial role in reproduction. Yet, seabirds spend most of their life at sea. They have developed a number of morphological, physiological and behavioural adaptations to forage in the marine environment. The use of acoustic signals at sea could potentially enhance seabirds’ foraging success, but remains largely unexplored. Penguins emit vocalisations from the sea surface when commuting, a behaviour possibly associated with group formation at sea. Still, they are unique in their exceptional diving abilities and feed entirely underwater. Other air-breathing marine predators that feed under water, like cetaceans, pinnipeds and marine turtles, are known to emit sound underwater, but such behaviour has not yet been described in seabirds. We aimed to assess the potential prevalence and diversity of vocalisations emitted underwater by penguins. We chose three study species from three different genera, and equipped foraging adults with video cameras with built-in microphones. We recorded a total of 203 underwater vocalisation from all three species during 4 h 43 min of underwater footage. Vocalisations were very short in duration (0.06 s on average), with a frequency of maximum amplitude averaging 998 Hz, 1097 Hz and 680 Hz for King, Gentoo and Macaroni penguins, respectively. All vocalisations were emitted during feeding dives and more than 50% of them were directly associated with hunting behaviour, preceeded by an acceleration (by 2.2 s on average) and/or followed by a prey capture attempt (after 0.12 s on average). The function of these vocalisations remain speculative. Although it seems to be related to hunting behaviour, these novel observations warrant further investigation.

## Introduction

Seabirds are highly vocal on land where acoustic communication often plays a crucial role in reproduction. While breeding, adults regularly commute between their foraging grounds at sea and their breeding colonies on land where they engage in nest care and chick provisioning. Every time they return to the colony, they must find and identify their partner and/or their offspring. In this context, acoustic signals are necessary for individual recognition (White & White, 1970; Charrier et al., 2001; Aubin & Jouventin, 2002; Curé, Aubin & Mathevon, 2011).

However, seabirds spend most of their time at sea. They have developed a number of morphological (Pütz et al., 1998; Weimerskirch et al., 2000), physiological (Nevitt, 2008; Angelier et al., 2008) and behavioural (Weimerskirch et al., 1994; Wakefield et al., 2013; Thiebault et al., 2016b) adaptations to forage in the marine environment. Their use of acoustic signals in this remote environment is poorly known. Recent studies have started to describe the use of aerial vocalisations in foraging seabirds. Gannets emit acoustically distinct vocalisations in different behavioural contexts when at sea, suggesting that each of these vocalisations convey distinct information (Thiebault et al., 2016a, 2019). Recent work has also shown that penguins emit vocalisations from the sea surface when commuting, a behaviour possibly associated with group formation and group foraging (Choi et al., 2017; McInnes et al., in press).

Penguins are unique among birds in their exceptional aquatic adaptations. They have lost the ability to fly but have developed extreme diving abilities (Williams & Williams, 1995). Using their modified wings for propulsion, they can perform serial dives to depths of 20–500 m in search of prey (Kooyman & Kooyman, 1995; Ropert-Coudert et al., 2006). A number of species have been observed to forage in groups (Norman & Ward, 1993; Takahashi et al., 2004; Copeland, 2008; McInnes et al., 2017), a behaviour in which vocal communication emitted from the sea surface could play a crucial role (Choi et al., 2017; McInnes et al., in press). Other air-breathing marine predators that feed under water, like cetaceans (Tyack & Clark, 2000), pinnipeds (Riedman, 1990) and marine turtles (Ferrara et al., 2017) are known to emit sound underwater, but such behaviour has not yet been described in seabirds.

In the current study, we aimed to assess the potential prevalence and diversity of vocalisations emitted underwater by foraging penguins. We chose three study species spanning three different genera–King penguins *Aptenodytes patagonicus*, Macaroni penguins *Eudyptes chrysolophus* and Gentoo penguins *Pygoscelis papua—*for the diversity of their vocalisations on land (Aubin & Jouventin, 2002; Searby, Jouventin & Aubin, 2004; Kriesell et al., 2018) and their diverse foraging ecology. King penguins dive to the lower limit of the photic zone generally between 100 m and 250 m (Pütz et al., 1998), where they feed mainly on myctophid fish (Adams & Klages, 1987). Macaroni penguins forage within the upper 100 m of the water column and predominantly target small crustaceans (Brown & Klages, 1987; Pichegru et al., 2011). In contrast to the former two species, Gentoo penguins tend to feed on a wide range of prey (Adams & Brown, 1989; Handley et al., 2017), in both pelagic and benthic habitat (Carpenter-Kling et al., 2017). We deployed video cameras with built-in microphones on foraging penguins of these three species to study their underwater vocal production. The behaviour of penguins was observed and quantified from video observations, and the vocalisations were analysed in the temporal and frequency domains.

## Materials and Methods

### Data collection

Fieldwork was conducted on penguins breeding at Marion Island, under a permit from the Nelson Mandela University Research Ethics Committee (Animal) (A14-SCI-ZOO-012/Extension). Deployments coincided with the brood phase of chick rearing; occurring August–September 2017 for Gentoo penguins, December 2017 for Macaroni penguins and February–March for King penguins. All species were sampled at Funk Bay (S 46°57.697′, E 37°51.518′), with Gentoo penguins additionally sampled at Bullard Beach (S 46°55.584′, E 37°52.949′) and Duikers Point (S 46°52.042′, E 37°51.423′).

Brooding adults were fitted with a modified Replay XD 1080 action camera (http://www.replayxd.com), housed within a custom aluminium tube pressure-tested to 300 m, for a total mass of 100 g and dimensions 104 × 26 × 28 mm. The cameras recorded footage at 1,920 × 1,080 p resolution, 30 frames s^−1^ and 120° field of view. They were further modified to include a flexible initial recording delay of up to 72 h, with recordings being split into six 15 min bins, each separated by 30 min. The camera recorded sounds at a 32 kHz sampling frequency with an internal microphone. The frequency response of this microphone was tested in a laboratory under water with the camera housed in the aluminium waterproof case. It measured to be 100–10.000 Hz (±14 dB). The cameras were deployed together with a combined resin-encased GPS (CatLog2; Catnip Technologies, Anderson, SC, USA; mass 30 g) logger and TDR (G5; CEFAS Technology Limited, England, UK; mass 2.7 g) for Gentoo and King penguins, or with a GPS-TDR-Accelerometer (Axy-Trek; Technosmart, Rome, Italy; mass 25 g) for Macaroni penguins. The total mass attached to penguins (including all devices and fastening materials) approximated 135 g for Macaroni penguins and 145 g for King and Gentoo penguins. The devices were secured to the plumage along the central line of the lower back (Fig. S1), placing the camera in such a way that the feeding behaviour of penguins was recorded in the field of view, while other devices were placed in a more caudal position in order to reduce drag and turbulence (Bannasch, Wilson & Culik, 1994). Previous studies have shown that similar deployment procedures have limited impact on penguin behaviour (Ballard et al., 2001) and subsequent breeding success (Agnew et al., 2013). Unfortunately, due to inconsistencies in recording and lack of adherence to pre-programmed schedules in the cameras, synchronisation of data between devices proved problematic. As a consequence, only data recorded from video cameras were used in this study.

**Figure 1:**
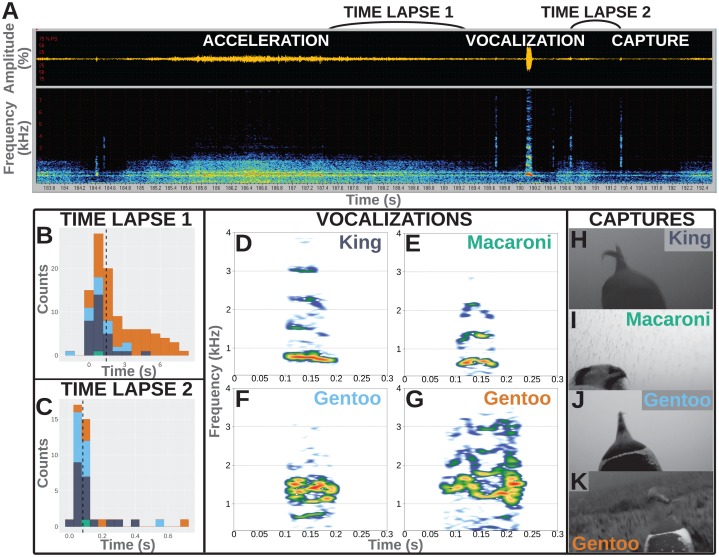
Underwater vocalisations in a hunting context. (A) Sound data including the oscillogram (i.e., amplitude over time) on top and the related spectrogram (i.e., frequency over time) just below, as displayed in Avisoft-SASLab Pro software. An acceleration is shortly followed with a vocalisation and then a prey capture attempt as observed on the video footage. (B) Distribution of the time lapse between the end of an acceleration and the start of a vocalisation (*N* = 104 in total, including one Macaroni, 29 King, 14 Gentoo pelagic and 60 Gentoo benthic vocalisations). (C) Distribution of time lapse between the start of a vocalisation and the prey capture attempt (*N* = 40 in total, including one Macaroni, 20 King, 13 Gentoo pelagic and six Gentoo benthic vocalisations). Histograms designed using the ‘ggplot2’ package in R (Wickham, 2016); dashed lines indicate the median values of the distribution. Colours on histograms relate to species and vocalisation context: dark blue, pelagic vocalisations by King penguins; green, pelagic vocalisation by Macaroni penguin; light blue, pelagic vocalisations by Gentoo penguins; orange, benthic vocalisations by Gentoo penguins. (D–G) Illustration of vocalisations emitted underwater by King, Macaroni and Gentoo (pelagic and benthic) penguins. All vocalisations chosen for illustration were observed to be immediately followed with a prey capture attempt. Spectrograms designed using the ‘Seewave’ package in R (Sueur, Aubin & Simonis, 2008), with Hamming function, FFT 512 points window size, 90% overlap. (H–K) Snapshots of prey capture attempts from video footage. Saturation, contrast and brightness of images were adjusted for better visualisation.

Deployed individuals were chosen based on the likelihood that the camera would start recording while the bird was at sea. For Gentoo and King penguins, we deployed devices in the afternoon and selected birds likely to depart the following day, estimated from time spent at the nest (assuming daily foraging trips for Gentoo penguins (Carpenter-Kling et al., 2017), and weekly trips for King penguins (Charrassin et al., 1998)). We performed Macaroni penguin deployments in the early morning, choosing females (discernible by relative bill size) from present pairs and assuming a same-day departure (Whitehead, 2017). Based on this, our cameras were set to start recording the same day, the following morning or after 3 days for Macaroni, Gentoo and King penguins respectively. Method of birds’ capture for deployment and retrieval depended on species. Both Gentoo and Macaroni penguins were captured by hand from the nest. For Gentoo penguins, exposed chicks were covered with cloth for the duration of the deployment or retrieval to prevent heat loss and predation. In six instances, returning Gentoo penguins were intercepted for device removal before their arrival at the nest. In this case, birds were caught using a telescopic pole with a crook on the end. Male Macaroni penguins remain at the nest for the duration of the brood phase. Male penguins therefore resumed nest duties while female Macaroni deployments and retrievals were being performed. For King penguins, individuals were not captured; only the bird’s head was covered to reduce stress. Device attachment and removal was conducted in place (i.e., while the bird continued to brood its chick). Devices were attached using overlapping layers of waterproof TESA^®^ tape (Beiersdorf AG, GmbH, Hamburg, Germany) with the ends fixed using cyanoacrylate glue (Loctite 401^®^). A cable-tie was fastened around the tape to further secure the units. The whole procedure was completed within 15 min. Following deployment, nests were checked daily for initial departure and returns. Devices were retrieved within 1 day of an individual’s return.

### Quantification of hunting behaviour

The behaviour of diving penguins was observed and quantified from video observations. All videos were annotated and analysed by a single person (AT). Footage was processed using the software Boris (Friard, 2019) and VLC media player (VideoLAN, Paris, France) so that the timing of each event of interest was recorded, using slow motion and frame-by-frame modes as necessary. Feeding dives were identified as those in which penguins dived straight down towards the depth, as opposed to performing shallow and directional commuting dives. Each dive was classified as pelagic when the penguin was moving exclusively in the water column, or benthic when the penguin was visibly feeding at the seabed. Prey capture attempts were identified as a jerky head movement (Videos S1–S3). They were classified as pelagic if they took place within the water column (during pelagic dives or during the descent or ascent of benthic dives), and benthic if they associated with the seabed. When prey items could be observed, they were identified as ‘crustacean’, ‘fish’ or ‘cephalopods’. Prey capture attempts were more easily observed when pelagic, with the penguin head moving upwards (towards the field of view of the camera), as opposed to when the penguin was browsing on the seabed with its head down. As a consequence, we assumed benthic prey capture to be largely underestimated. For this reason, the positions of prey captures within each dive were recorded only for pelagic dives, provided the recording started in the early stages of the dive (before or at the very beginning of the descent). Conspecifics in the vicinity of the equipped bird were also recorded (underwater during a dive or at the sea surface just before or after a dive). We acknowledge the fact that in some cases conspecifics could have been present but not observed in the limited field of view of the camera.

Furthermore, the hunting technique of penguins often involves prey pursuit (Ropert-Coudert et al., 2000). A number of associated accelerations (or ‘dashes’, Ropert-Coudert et al., 2000) were observed during the feeding dives and were used as a proxy for prey capture attempts (when they could have occurred outside of the field of view of the cameras). Accelerations were observed in both the video and the spectrograms extracted from the sound recorded on cameras. However, the timing of accelerations was more easily identified from the spectrograms (Fig. 1A).

### Acoustic measurements on vocalisations

Sound data were extracted from the camera recordings. They were resampled at 16 kHz as no vocalisation was observed to contain energy at frequencies higher than 7 kHz. All the vocalisations were analysed using Avisoft-SASLab Pro (version 5.2.13; Avisoft Bioacoustics, Glienicke/Nordbahn, Germany). The spectrogram of each recording (Hamming function, FFT 512 points window size, 75% overlap) was visualised over a sliding window of 10 s length to identify and label all the vocalisations. They were selected for frequency measurements wherever the quality of the recordings allowed (i.e., low background noise, no overlap with another sound).

The recording devices were first aimed at collecting behavioural data on the foraging activities of penguins, but because the cameras included a built-in microphone, we also had the opportunity to study penguin vocalisations at sea. However, the quality of the frequency response of the microphone (camera fitted in an aluminium waterproof case) was not sufficient for an exhaustive acoustic analysis. As a consequence, to give the best possible general description of these underwater vocalisations, given the data, we chose only three acoustic parameters for which we were confident of the accuracy of their measurements. These included the duration of the vocalisation (DurCall, s) measured on the oscillogram, the fundamental frequency (F0, Hz) and the frequency of maximum amplitude (Fmax, Hz) both measured on the frequency spectrum.

### Statistics

All analyses were conducted in R software (R Core Team, 2019). The distributions of variables were tested for normality using the Shapiro–Wilk test. Since the hypothesis of normality was rejected for most data, the median and variance of distribution among groups were compared using non-parametric tests, the Fligner–Killeen test of homogeneity of variance and the Kruskal–Wallis rank sum test, respectively. Results are shown as mean ± standard deviation.

## Results

We recorded a total of 10 h 14 min 43 s of footage showing penguins at sea, among which a total of 93 dives (not commuting) were recorded for all three species for an accumulated duration of 4 h 43 min 26 s underwater (Table 1). From this footage, 26 feeding pelagic dives were recorded from six King penguins, 13 from two Macaroni penguins, and a mix of 54 pelagic and benthic dives were recorded from 12 Gentoo penguins. King penguin feeding dives were the longest, lasting 4.9 ± 0.9 min. They comprised between zero and 22 prey capture attempts, with a total of 114 attempts observed across 13 feeding dives. When prey items were observed, they were all fish. Conspecifics were observed during nine of the 26 King penguin feeding dives, either on the sea surface before or after a dive, or underwater showing unsynchronised diving behaviours (i.e., the study penguin is descending while a conspecific is ascending).

**Table 1:** Penguin dives as observed from bird-borne video cameras. Summary of the dives (not commuting) observed from video cameras deployed on penguins. Duration of footage only includes parts where penguins were diving. Dives were classified as pelagic if the penguin was moving exclusively in the water column, or benthic if feeding on the seabed. Prey capture attempts were identified as a jerky head movement, and were most probably underestimated in benthic dives (due to the limited field of view of the camera). Conspecifics were observed in the vicinity either underwater water or at the sea surface just before or after a dive. *N*, number of measured vocalisations; SD, standard deviation.

Species	Dive type	Duration of diving footage	*N* dives (*N* individuals)	Duration of dives (min)			Conspecifics	Vocalisations		Prey capture attempts		
				*N* dives complete	Mean ± SD	Range	*N* dives	*N* dives (*N* individuals)	*N* vocalisations per dive phase	*N* dives	*N* captures	Prey type
King penguin	Pelagic	1 h 37 min 39 s	26 (6)	11	4.9 ± 0.9	(4.0–7.4)	9	10 (2)	5 Descent	13	114	Fish
									29 Bottom			
									0 Ascent			
Macaroni penguin	Pelagic	25 min 42 s	13 (2)	8	2.2 ± 0.3	(1.7–2.6)	7	1 (1)	0 Descent	10	155	Crustaceans
									1 Bottom			
									0 Ascent			
Gentoo penguin	Pelagic	54 min 17 s	23 (8)	10	2.7 ± 0.7	(1.5–3.6)	0	6 (4)	2 Descent	17	352	Fish, crustaceans, cephalopods
									10 Bottom			
									0 Ascent			
Gentoo penguin	Benthic	1 h 45 min 47 s	31 (9)	15	4.2 ± 0.5	(3.2–5.2)	0	20 (6)	35 Descent	22	74	Fish, crustaceans
									108 Bottom			
									13 Ascent			

Macaroni penguin dives were the shortest, lasting 2.2 ± 0.3 min (Table 1). They comprised between zero and 74 prey capture attempts, with a total of 155 attempts observed across 10 feeding dives. Both Macaroni penguins fed on schooling krill. Conspecifics were observed during seven of the 13 Macaroni penguin feeding dives, either on the sea surface before or after a dive, or underwater showing synchronised diving behaviours (i.e., penguins are descending and surfacing together).

Of the 54 Gentoo penguin feeding dives, 23 were classified as pelagic and 31 as benthic. Pelagic dives lasted on average 2.7 ± 0.7 min and comprised between zero and 69 prey capture attempts, with a total of 352 attempts observed across 17 dives (Table 1). Benthic dives were longer, lasting 4.2 ± 0.5 min, with only 74 prey capture attempts observed across 22 dives (possibly limited by the field of view of the camera looking forward, while the penguin’s head was facing downward). Gentoo penguins fed on fish, cephalopods and small crustaceans. No conspecifics were observed in the surroundings of any of the Gentoo penguin feeding dives.

### Behavioural contexts of underwater vocalisations

A total of 203 underwater vocalisations were recorded: 34 from two King penguins, a single one from a Macaroni penguin and 168 from Gentoo penguins (60 classified as pelagic vocalisations and 108 as benthic vocalisations). Based on the camera footage, penguins were mostly solitary while vocalising underwater. Only five of all vocalisations, all from King penguins, were emitted in feeding dives where conspecifics were observed. The underwater vocalisation recorded from a Macaroni penguin was emitted in a dive with no conspecifics, while no vocalisations were recorded in another dive where synchronised diving behaviour with conspecifics was observed. Conspecifics were never observed in the surroundings of Gentoo penguins.

All vocalisations were emitted during feeding dives, mostly during the bottom phase of the dives (148/203 vocalisations vs 42 during the descent and 13 during the ascent). More than 50% of the recorded vocalisations were directly associated with a hunting behaviour: immediately following acceleration (supposedly chasing prey) and/or immediately followed by a prey capture attempt. Not all vocalisations were preceded with an acceleration, and not all accelerations were followed with a vocalisation. Accelerations preceded vocalisations in 104 (60 benthic vocalisations and 44 pelagic vocalisations) out of 203 cases, and lasted 6.2 ± 4.4 s (range 0.5–17.0 s). Those included one vocalisation from a Macaroni penguin, 29 from King penguins, 14 and 60 from Gentoo penguins in a pelagic and benthic context, respectively. The accelerations preceded the vocalisations by 2.2 ± 2.1 s, with two vocalisations emitted within the last 2 s of the acceleration (range −1.6 to 8.4 s, Fig. 1A). Within the limited field of view of the camera, we observed 40 vocalisations to be immediately followed with a prey capture (others could have been missed if outside of the field of view). Those included one vocalisation from a Macaroni penguin, 20 from King penguins, 13 and six from Gentoo penguins in a pelagic and benthic context, respectively. The time lapse between the start of a vocalisation and prey capture averaged 0.12 ± 0.13 s (range 0.02–0.68 s, Fig. 1C). For 30 of these 40 prey capture attempts the prey could be identified: four as crustaceans (1 + 3 hunted by Macaroni and Gentoo penguins respectively) and 26 as fish (16 + 10 hunted by King and Gentoo penguins respectively). Based on the entire number of prey capture attempts, only a small proportion of them were preceded with a vocalisation, and this varied greatly with the prey type: <1% for hunted crustaceans (4/463) vs 19% for hunted fish (26/134). In the case of fish (enabled through sufficient data), vocalisations were more likely at the first (67%, 8/12) vs following (10%, 9/87, range per position 0–33%) prey capture attempts within a dive.

### Acoustics of underwater vocalisations

Recorded vocalisations were very short in duration, lasting 0.06 s on average (Table 2), and did not vary much between species (Kruskal–Wallis chi-squared = 1.530, df = 1, *p*-value = 0.216; Fligner–Killeen chi-squared = 1.047, df = 1, *p*-value = 0.306; *N* = 34 King + 168 Gentoo). Calls showed a harmonic structure and the values of the fundamental frequency, F0, averaged 500–600 Hz for all species (Table 2) with no significant differences in distributions between species (Kruskal–Wallis chi-squared = 2.157, df = 1, *p*-value = 0.142; Fligner–Killeen chi-squared = 0.029, df = 1, *p*-value = 0.865; *N* = 34 King + 168 Gentoo). The frequency of highest energy, Fmax, averaged 998 Hz for King penguins and 1,097 Hz for Gentoo penguins, and their distribution significantly varied in median (Kruskal–Wallis chi-squared = 4.280, df = 1, *p*-value = 0.039; *N* = 34 King + 168 Gentoo). Notably, one Gentoo individual performed whistle calls (seven whistles recorded during two successive feeding dives, Video S3). The single vocalisation recorded from a Macaroni penguin was emitted with a lower Fmax at 680 Hz (Table 2).

**Table 2:** Penguins underwater vocalisations. Summary of the distribution of acoustic variables measured on underwater vocalisations. DurCall, duration of the vocalisation (s); F0, fundamental frequency (Hz); Fmax, frequency of maximum amplitude (Hz); *N*, number of measured vocalisations; SD, standard deviation.

Acoustic variables	King penguin Pelagic vocalisations			Macaroni penguin Pelagic vocalisation		Gentoo penguin Pelagic vocalisation			Gentoo penguin Benthic vocalisation		
	*N*	Mean ± SD	Range	*N*	Value	*N*	Mean ± SD	Range	*N*	Mean ± SD	Range
DurCall (s)	34	0.06 ± 0.03	(0.02–0.18)	1	0.05	60	0.07 ± 0.05	(0.02–0.33)	108	0.05 ± 0.03	(0.02–0.18)
F0 (Hz)	23	535 ± 169	(309–850)	1	697	27	628 ± 418	(139–1539)	64	475 ± 249	(140–1441)
Fmax (Hz)	28	998 ± 389	(648–1980)	1	680	39	1136 ± 413	(625–2011)	81	1078 ± 354	(480–1890)

## Discussion

Penguins are known for their remarkable diving abilities. Our study further demonstrates their aquatic abilities and adaptation to the marine environment. Each of the three species studied here is classified in a different genus of the Spheniscidae family and exhibits varied foraging behaviour (Adams & Klages, 1987; Brown & Klages, 1987; Adams & Brown, 1989). Yet, all studied species vocalised under water in the various feeding contexts. This suggests that such underwater vocal behaviour may exist in all penguin species. However, underwater vocalisations were recorded in much higher proportion when penguins were feeding on fish, compared to crustaceans or cephalopods. As a consequence, underwater vocalisations may be expected to be more common in piscivorous penguins.

### The production of sound under water

As the first record of underwater vocalisations in seabirds, these observations raise a number of questions regarding the emission of such sounds. How are penguins able to produce sound at deep depth, given the high pressure of the water? In saltwater, the pressure would vary between approximately 1,100,000 Pa (11 bar) at 100 m and 3,600,000 Pa (36 bar) at 350 m depth. Penguins must have physiological and anatomical adaptations to prevent their trachea from collapsing when diving. They feed and ingest prey under water, so their trachea must also be resistant to the passing of food through the oesophagus at high pressure. One possible adaptation could be the septum trachealis medialis (STM) which medially divides the trachea. STM was described in another marine species feeding under water, the leatherback turtles (Davenport et al., 2014), but also exists in King penguins (H. Kriesell, T. Aubin, 2019, personal communication). The STM contains ossified plates in its caudal third and may play a vital role in preventing the compression of the trachea while penguins ingest prey or emit sounds at deep depth.

Another question to be addressed is whether these sounds were emitted intentionally by the penguins or, instead, could they be mechanistically released by a breath-holding diving predator? Among all recorded dives, vocalisations were recorded exclusively when prey captures were also observed in the same dive and mostly during the bottom phase of the dives (when prey captures occur the most often). This shows a strong association between underwater vocalisations and hunting behaviour and suggests that the sounds were not passively produced but rather controlled to be emitted in specific situations. In addition, vocalisations were recorded in combination of only a small proportion of the observed prey captures and a small proportion of accelerations, suggesting they were not mechanistically emitted as a consequence of increased movement or every time a penguin opened its beak under water. Vocalisations furthermore did not have structures similar to noise or pulse but displayed clear harmonic structures, sometimes with frequency and amplitude modulations (Fig. 1D–1G). As a consequence, the recorded vocalisations seemed to be produced under control.

Now, to assess whether sound production is intentional and associated with a specific function remains a challenge. Below we propose and develop some hypotheses. Vocalisations could simply be an expression of excitement of finding food. Or else, they could fulfil physiological needs related to diving and feeding in apnoea. Finally, they could have a function for social communication or for capturing prey.

### Underwater vocalisations as a by-product of physiological needs?

Penguins have physiological adaptations for diving, with lungs and air sacs capacities higher than allometric predictions (Ponganis, Leger & Scadeng, 2015). They can control their buoyancy (i) by adjusting the amount of air inhaled prior to a dive depending on the depth of the expected dive and (ii) by exhaling air during the ascent to slow down their speed (Sato et al., 2002). Accordingly, in two instances we observed a Gentoo penguin exhaling air, and thus emitting sound, during the last part of the ascent phase. However only 6% of vocalisations were recorded during the ascent of dives showing that this mechanism was rarely used, contrary to what has been reported in seals (Hooker et al., 2005). Vocalisations were most often recorded during the bottom phase of dives, where the pressure is highest and buoyancy should be negligible (Sato, Watanuki & Naito, 2006). Alternatively, vocalisations could result from the occasional need to expel an air bubble from the trachea in order to be able to ingest prey under water.

### Underwater vocalisations for social communication?

Some species of penguins have been observed feeding in groups (Norman & Ward, 1993; Takahashi et al., 2004; McInnes et al., 2017), a behaviour in which vocalisations emitted from the sea surface can play a role (Choi et al., 2017; McInnes et al., in press). Vocalisations emitted under water could be used to further coordinate or synchronise feeding activities. The limit to this hypothesis is that we did not record underwater vocalisations concomitantly to synchronised diving activity (even when such activity was recorded). As a consequence, it seems unlikely that these vocalisations could have been used to coordinate feeding activities. However, we cannot exclude the possibility of penguins being present in looser aggregations and making use of underwater acoustic cues. The hearing abilities of penguins is not yet known, although penguins are known to react to underwater sounds (Pichegru et al., 2017). Studies on other diving birds, like cormorants or sea ducks, have shown that they can hear underwater despite an in-air adapted ear (Therrien, 2014; Johansen et al., 2016). In this context, the vocalisations emitted at the first prey encounter within a dive could inform conspecifics of the presence of prey, as well as its localisation. Indeed, animals can develop extreme abilities to locate sound by ear (Griffin, 1958; Schusterman et al., 2000). Since sound travels much further than light under water and visual cues are limited at depth, the vocalisations emitted by penguins when capturing prey could be used as acoustic cues for locating feeding conspecifics.

### Underwater vocalisations for capturing prey?

The fact that underwater vocalisations were clearly associated with feeding and hunting behaviour raises the question of the adaptive value of this behaviour. Since only a small proportion of the observed capture attempts were preceded with a vocalisation, they cannot be a prerequisite for efficient prey capture, but rather are probably used in some specific situations. Vocalisations could potentially be used in response to a prey escaping or showing avoidance behaviour (Handley et al., 2018). For example, in one instance we recorded a King penguin successively emitting three vocalisations in what seemed like a repeated prey capture attempt (Video S4). Most fishes have hearing abilities ranging between 30 and 3,000 Hz (Popper & Schilt, 2008). Similarly, the hearing abilities of various species from the Order Decapoda (classified in the Superorder Eucarida, together with Euphausiacea) is situated between 100 and 3,000 Hz (Popper, Salmon & Horch, 2001). Those hearing values fall within the range of production of penguin underwater vocalisations. The ability for the prey to receive the sound wave also depends on the pressure and particle motion of the vocalisation (Radford et al., 2012). But because they were emitted from such a short distance (0.1 s before capture), we can assume that they could be heard or felt (vibration) by the prey. In a situation where the penguin has come so close to the prey, but the prey is about to escape, a vocalisation or a vibrational wave might be enough to startle the prey (as shown in herrings Nestler et al., 1992) and immobilise it for a split second, just enough to allow prey capture. The ability of marine mammals to stun prey using sounds has long been hypothesised and debated (Norris & Mohl, 1983; Marten et al., 2001; Benoit-Bird, Au & Kastelein, 2006; Fais et al., 2016). In particular, some specific sounds emitted by dolphins over low frequencies (most energy under 5 kHz, so more similar to what we recorded from penguins) can disorientate or change the behaviour of the prey, if not stun them (Marten et al., 2001).

## Conclusion

We have here provided the first observations of underwater penguin vocalisations while foraging at sea. As intriguing as these observations are, we failed to demonstrate the adaptive significance of this behaviour, although it seems likely to enhance foraging success. Our study was restricted by the quality of sound recordings and the limited field of view of mounted cameras. We strongly encourage further research on this intriguing behavioural phenomenon, which contributes to the debates on the uses of underwater vocalisations by diving predators.

## Supplemental Information

10.7717/peerj.8240/supp-1 Supplemental Information 1 Acoustic measurements on underwater vocalisations by penguins.

10.7717/peerj.8240/supp-2 Supplemental Information 2 Dives (no commuting) from penguins.

10.7717/peerj.8240/supp-3 Supplemental Information 3 Prey capture attempts from penguins as observed from bird-borne video cameras.

10.7717/peerj.8240/supp-4 Supplemental Information 4 Gentoo penguin with devices attached along its back. Photo credit: Paige Green.

10.7717/peerj.8240/supp-5 Supplemental Information 5 King penguin feeding on fish, as observed from onboard video camera. A series of five prey capture attempts can be observed, with a vocalisation emitted just before the second capture attempt.

10.7717/peerj.8240/supp-6 Supplemental Information 6 Macaroni penguin feeding on schooling krill, as observed from onboard video camera. A series of four prey capture attempts can be observed, with a vocalisation emitted just before the first capture attempt.

10.7717/peerj.8240/supp-7 Supplemental Information 7 Gentoo penguin feeding on small prey, as observed from onboard video camera. A series of seven prey capture attempts can be observed, of which six are preceded with a vocalisation.

10.7717/peerj.8240/supp-8 Supplemental Information 8 King penguin feeding on fish, as observed from onboard video camera. The penguin emits a series of three successive vocalisations in a prey capture attempt.

## Additional Information and Declarations

### Data Availability

The following information was supplied regarding data availability: Datasets are available in the Supplemental Files.
