# Supplementary figures and images for: First evidence of underwater vocalisations in hunting penguins
Source: PeerJ. 2019 Dec 18;7:e8240. doi: 10.7717/peerj.8240 (PMC6966993; doi:10.7717/peerj.8240)

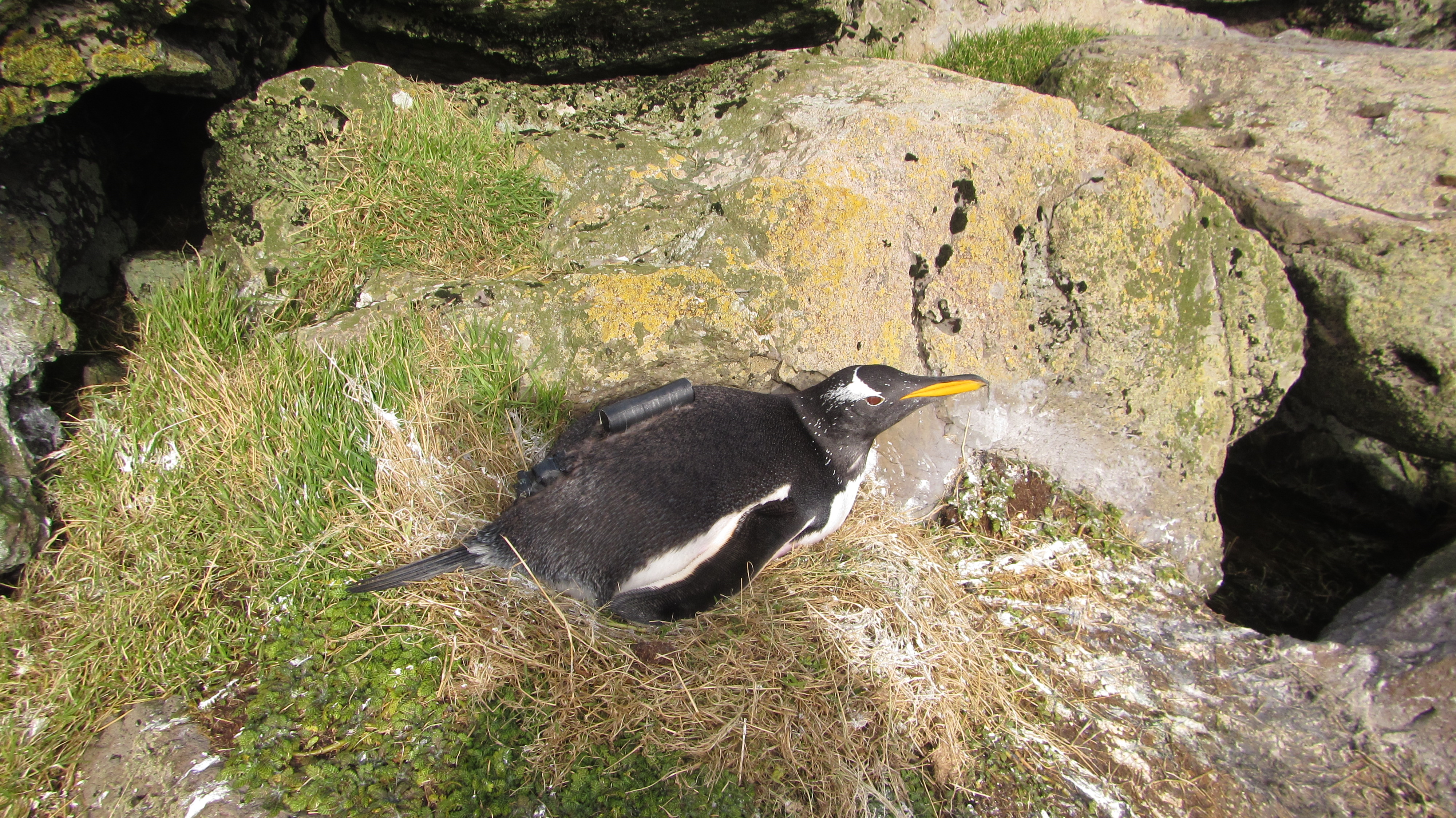

Supplement: Supplemental Information 4 — Photo credit: Paige Green. [file peerj-07-8240-s004.png]
